# The Management of Medullary Thyroid Carcinoma in the Era of Targeted Therapy

**DOI:** 10.17925/EE.2016.12.01.39

**Published:** 2016-03-15

**Authors:** Brian Ng-Cheng-Hin, Kate L Newbold

**Affiliations:** 1. NIHR Royal Marsden Hospital and Institute of Cancer Research BRC, London, UK.

**Keywords:** Medullary thyroid cancer, targeted therapy, kinase inhibitors

## Abstract

Medullary thyroid cancer (MTC) is a rare cancer comprising approximately 5% of all thyroid cancers. The majority arises sporadically but around 25% are hereditary forming part of the Multiple Endocrine Neoplasia (MEN) type 2 syndromes. The initial management is surgical, the extent of resection determined by radiological stage, presence of and specific REarranged during Transfection (RET) oncogene mutation and level of serum calcitonin. External beam radiotherapy may be utilised in the adjuvant setting to improve local control rates. Conventional cytotoxic agents remain essentially futile in the management of advanced MTC with response rates of around 15–20% at best. Over the last decade, alongside a greater understanding of the molecular pathogenesis of MTC we have seen the development of small molecule agents including tyrosine kinase inhibitors targeting vascular endothelial growth factor receptors (VEGFRs) and RET with activity in advanced MTC. This review will examine the evidence for this therapeutic approach, when to consider initiating and how to manage toxicities arising from such therapies in the treatment of advanced MTC.

Medullary Thyroid Cancer (MTC) is a rare cancer of the thyroid parafollicular or ‘C’ cells, and is estimated to make up around 5–10% of all thyroid cancers.^1^ MTC was first described in 1906 by Jacquet as a thyroid tumour with amyloid, but the term MTC and its histological description of a solid non-follicular tumour with amyloid features was coined by Hazard et al., in 1959.^2,3^ Parafollicular C cells originate from the neural crest and so in keeping with their neuroendocrine origin, MTC secretes calcitonin (Ct), serotonin, chromogranin A and expresses carcinoembryonic antigen (CEA). Serum levels of Ct and CEA are used as tumour markers to aid diagnosis, monitor disease progression and assess response to treatment.

75% of MTCs are sporadic and the remaining 15% are hereditary as part of the Multiple Endocrine Neoplasia (MEN) type 2 syndrome and the familial MTC (FMTC).^4,5^ The MEN syndrome arises from a germline mutation in the REarranged during Transfection (RET) oncogene. The MEN type 2 syndromes can be subcategorised into MEN2A and MEN2B according to clinical features. MEN2A is comprised of MTC, hyperparathyroidism and phaeochromocytoma whereas MEN2B consists of MTC, phaeochromocytoma, mucosal neuromas and a marfanoid habitus. FMTC is thought to be part of the spectrum of MEN2A. Patients have FMTC when they harbour a germline RET mutation but do not display the other phenotypic characteristics of MEN2A.

Patients may present with a solitary thyroid nodule or with palpable cervical neck nodes. Symptoms can result from pressure effects of the disease such as dysphagia, dyspnoea, hoarseness and coughing. Ct secretion by the tumour may cause systemic symptoms such as flushing and diarrhoea. Not uncommonly, on direct questioning, patients will have suffered from irritable bowel symptoms for years. At diagnosis, lymph nodes metastases are found in around 60–70%, with 7–20% having distant metastatic disease and over 20% will die from their metastatic disease.^6–8^

Overall, the prognosis of MTC is good but is dependent on the stage; with reported 10-year overall survival of 95% with localised disease, 75% with regional disease and falling to 40% in those presenting with distant metastases.^7^

## Current Standard Management

### Early Stage Medullary Thyroid Cancer

Treatment of localised disease is surgical with total thyroidectomy and selective lymphadenectomy. Thorough pre-operative staging together with serum Ct levels are critical to the decision making for the extent of neck dissection and lymphadenectomy required. Debate continues as to the role and extent of elective dissection of the radiologically negative neck. Patients with distant metastases or serum Ct levels suggestive of distant metastatic disease may be offered a more conservative local surgical approach. The American Thyroid Association guidelines provide useful background for these decisions.^8^

Carriers of a RET germline mutation should be offered preventative surgery with total thyroidectomy. The age at which this is recommended in gene carriers, depends on the level of risk conferred by their particular mutation.^8^ The level of serum Ct also aids the decision of when to consider surgery.^8^ These patients and their families should be managed within an expert team comprising of endocrinologists (with paediatric expertise), geneticists, paediatric thyroid surgeons and oncologists where appropriate.

In a large series of cases of 899 patients with MTC with data collected between 1952 and 1996, surgery normalised Ct and CEA levels (biochemical cure) in 43% and of these only 9% developed recurrent disease.^9^ Biochemical cure post initial surgery remains the best prognostic marker leading to a 10-year survival of 97.7%.^9^ This drops to 70.3% if biochemical cure is not achieved.^9^ More recently the response post initial treatment has been shown to be a better predictive marker than TNM (Tumour Node, Metastasis classification of malignant tumours) staging with 88%, 4% and 0% of patients in groups of excellent response (both biochemical and structural), biochemical incomplete response, and structural incomplete response respectively attaining a status of no evidence of disease.^10^

### Advanced Stage Medullary Thyroid Cancer

In the presence of incurable locally advanced and metastatic disease, the decision on the timing of further therapies rests on the rate of disease progression, the presence of symptoms and the potential side effects of the proposed local or systemic treatment intervention.

A number of treatment modalities have a role in the treatment of progressive MTC. Localised ablative therapies such as surgery radiotherapy, radiofrequency ablation or embolisation should be considered for well-defined deposits of disease that are causing or likely to cause specific symptoms. These modalities can improve symptoms caused by tumour bulk such as pain or critical organ obstruction but may also reduce systemic symptoms if Ct production falls as a result.^8,11^ External beam radiotherapy (EBRT), including advanced delivery techniques such as stereotactic or intensity modulated (IMRT), has a specific role in the palliation of symptoms from brain and bone metastases.

Around 40% of MTC expresses somatostatin receptors and this has been explored as potential therapeutic target.^12^ Although somatostatin receptor analogues have not been shown to influence survival in MTC, they may relieve systemic symptoms such as flushing and diarrhea.^13^ Conventional chemotherapy agents have largely been unsuccessful with low response rates of 15–20%.^14–16^

These treatment interventions however have been ineffective in prolonging survival in the advanced disease setting. In the era of small molecular inhibitors and with understanding of the aberrant signaling pathways leading to the development of MTC, there has been significant interest in finding small molecule inhibitory targets.

## Background on Mechanism of Tyrosine Kinase Inhibitors and Relevance to Biology of Medullary Thyroid Cancer

Tyrosine Kinase Receptors (TKRs) on the cell surface are activated by specific ligands; this activation leads to a cascade of autophosphorylation and to the activation of downstream signaling pathways towards events such as cell growth.

The RET proto-oncogene was discovered by Takahashi et al. in 1985.^17^ The RET proto-oncogene encodes a tyrosine kinase transmembrane receptor and is mutated in virtually all hereditary MTC and in around 50% of sporadic MTC.^18^ The M918T mutation is present in 85% of somatic RET mutations and has been associated with a poorer prognosis and more aggressive clinical course.^19^ A mutated RET leads to a constitutively active protein receptor with constant growth signals to the cell. It is unclear whether RET is the initiator of oncogenesis or whether it acts as a driver once MTC has been initiated. A proportion of MTC without a RET mutation shows mutations in downstream signaling pathways such as in GTPase proteins and enzymes such as RAS, mammalian target of rapamycin (mTOR) and phosphoinositide 3-kinase (PI3K).^20^

Other signaling pathways that have been implicated in the growth and invasiveness of MTC are the epidermal growth factor receptor (EGFR), the vascular endothelial growth factor receptor (VEGFR) and the hepatocyte growth factor receptor (c-MET) pathways.^21–23^ Therefore, targeting these receptors has also been of interest as a way of inhibiting or blocking these aberrant signaling pathways. Many of these signaling pathways are inter-linked which may explain the development of resistance and partial rather than complete responses to drugs.

## Tyrosine Kinase Inhibitors

A tyrosine kinase inhibitor (TKI) blocks the receptor signaling by preventing the autophosphorylation of the receptor and hence the downstream activation signaling pathways. TKIs tend to be multikinase inhibitors as they inhibit many TKRs. Two such multikinase inhibitors currently approved for first line systemic therapy in advanced or metastatic MTC are vandetanib and cabozantinib.

### Vandetanib

Vandetanib is a multikinase inhibitor of RET, VEGFR 2, 3 and EGFR. Vandetanib is an oral tablet, which is taken daily. Phase II data showed that Vandetanib was an active agent in MTC.^24^ The maximum tolerated dose was 300 mg/day and in the 30 patients with hereditary MTC, 16 patients had stable disease lasting longer than 24 weeks and 6 patients had confirmed partial responses. A second phase II trial of 19 patients with hereditary MTC showed partial response in 3 and stable disease in 10 patients lasting longer than 24 weeks.^25^

The United States Food and Drug Administration (FDA) and the European Medicines Agency (EMA) both approved the use of vandetanib in April 2011 and November 2011 respectively following the demonstration of the therapeutic efficacy of vandetanib in a phase III trial, the ZETA trial.^26^ This trial was an international randomised, placebo-controlled, double-blind phase III trial that randomised patients to either placebo or a starting dose of 300 mg per day of vandetanib. Three hundred and thirty-one patients with advanced or metastatic hereditary or sporadic MTC were randomised in a 2:1 ratio to receive vandetanib or placebo. The patients on placebo, who progressed, were unblinded and allowed to crossover to the vandetanib arm. Of the 13 patients with an objective response initially assigned to placebo, 12 of the observed objective responses were when they were switched to the open-label vandetanib.

At the time of data cut-off, the median progression free survival (PFS) of the vandetanib arm was not reached, but its predicted value was calculated to be 30.5 months. This compares to 19.3 months in the placebo arm and the improvement was statistically significant. As a RET mutation drives the majority of MTC, this trial had planned to analyse whether the RET mutation status would influence PFS and response. However, this question remains unanswered as the subgroup analysis was inconclusive due to small number of RET negative patients and a large number of patients with unknown mutation status.^26^

Patients may remain on vandetanib for a significant amount of time with the median being 90.1 weeks in the ZETA study. Within the study 12% of patients on vandetanib stopped treatment due to an adverse event. Of those who remained on vandetanib, 35% required a dose reduction due to significant side effects. Side effects such as diarrhoea, nausea, rash and hypertension occurred in more than 30% of patients due to vandetanib. Prolongation of QTc interval on the electrocardiogram (ECG) occurred in 35% of patients, and this requires careful monitoring as development into Torsade de Pointes can be life threatening. Furthermore, grade 3 adverse events were more common in patients receiving vandetanib with 11% having diarrhoea, 9% hypertension and 8% prolongation of QTc interval. Vandetanib also led to 49.3% of patients needing an increase dose of thyroxine compared to 17.2% in the placebo arm.^26^

### Cabozantinib

Cabozantinib is a multikinase inhibitor that targets c-MET, VEGFR2 and RET pathways in MTC. Its activity was first demonstrated in a phase I/II trial in which 17 out of 35 patients had a partial response.^27^

The FDA and EMA approved its use in patients with advanced MTC following the publication of a phase III trial, the EXAM trial.^27^ This trial was an international, double-blind, randomised, placebocontrolled phase III clinical trial that randomised 330 patients to either cabozantinib or placebo in a 2:1 ratio. This study showed a statistically significant improvement in median estimated PFS to 11.2 months in the cabozantinib arm compared to 4 months in the placebo arm. RET mutation-positive and -negative subgroups demonstrated similar response rates to cabozantinib (32% and 25%, respectively).^28^ Although the final analysis of overall survival did not show a statistically significant difference between those patients receiving placebo and those receiving cabozantinib, a subsequent retrospective subgroup analysis of the patients with a germline or somatic RET M918T mutation had an overall survival of 44.3 months on cabozantinib versus 18.9 months on placebo (hazard ratio 0.60).^29^

As with vandetanib, patients on cabozantinib experience side effects, which include diarrhoea, hypertension, palmar-plantar erythrodysesthesia, fatigue and occasionally gastrointestinal fistulation. Cabozantinib is given orally at a starting dose of 140 mg, but in the EXAM trial 79% needed a dose reduction and 16% stopped the drug due to significant toxicities. Of patients on cabozantinib, 69% reported grade 3 or 4 adverse events mainly diarrhoea, palmar-plantar erythrodysesthesia and fatigue. Similar to vandetanib, cabozantinib caused a raised level of thyroid stimulating hormone (TSH) in 57% requiring an increase in thyroxine dose.^28^ This may be related to an increase in activity of type 3 deiodinase.^30^ Other significant adverse events more common in the cabozantinib group compared to placebo were mucosal inflammation, hypocalcaemia, pulmonary embolism and hypertension. Gastrointestinal perforations, fistula development, and hemorrhage occurred in the cabozantinib arm of this study. These events are thought to be related to inhibition of the VEGF pathway. Although low frequency these are clearly potentially life-threatening adverse events and caution is required when selecting patients for therapy, particularly those patients who are at risk for such events with tracheal or oesophageal invasion and perhaps due to extensive prior treatment.^28^ Cabozantinib and vandetanib have not been directly compared in a clinical trial so superiority of one over the other is not known. *Table 1* highlights the key findings in both phase III studies. When deciding which drug to initiate (if the clinician has the choice of more than one available drug) the adverse event profile of each agent together with the patient’s co-morbidities, concomitant medications and impact of the site of disease in the context of the relative contraindications should all be taken into account.

**Table 1:** Comparison of Key Features in the Phase III Studies in Advanced Medullary Thyroid Cancer

	Vandetanib (ZETA)^26^	Cabozantinib (EXAM)^28,29^
Targets inhibited	RET, VEGFR2,3, EGFR	c-MET, VEGFR2, and RET
Trial design	Randomised controlled trial (2:1 active drug:placebo)	Randomised controlled trial (2:1 active drug:placebo)
Number	331	330
Eligibility criteria	No requirement for Progressive disease	RECIST confirmed Progressive disease within 14 months
Age at enrollment interventional arm	50.7 years (mean)	55 years (median)
RET mutation status in interventional arm	Positive: 59% Negative: 1% Unknown: 40%	Positive: 46.1% Negative: 14.2% Unknown: 39.7%
Prior systemic therapy	39%	37%
Prior TKI therapy	Unknown	20.1%
Median PFS (active versus placebo)	30.5 months versus 19.3 months	11 months versus 4 months
PFS hazard ratio	0.46; 95% CI, 0.31 to 0.69; p<0.001	0.28; 95% CI, 0.19 to 0.40; p<0.001
Objective Response Rate	45% versus 13%	28% versus 0%
Overall Survival	Not reported	27 months versus 21 months
Overall survival in RET positive	Not reported	44.3 months versus 18.9 months

Cl = confidence interval, c-MET = hepatocyte growth factor receptor, EGFR = epidermal growth factor receptor, PFS = progression free survival, RECIST = Response Evaluation Criteria in Solid Tumors, RET = rearranged during transfection, TKI = tyrosine kinase inhibitor, VEGFR = vascular endothelial growth factor receptor.

## Other Targeted Agents

Lenvatinib is a multikinase inhibitor that inhibits VEGFR 1– 3, fibroblast growth factor receptor (FGFR) 1–4, platelet derived growth factor receptor (PDGFR), and RET. It is currently licensed for treatment of iodine refractory differentiated thyroid cancers following publication of a prospective randomised controlled trial comparing lenvatinib to placebo in this group of patients (the SELECT trial).^31^ Schlumberger et al., reported a phase II study of lenvatinib in MTC.^32^ They reported a disease control rate of 80%, an objective response rate of 36% and median PFS of nine months. Similar to vandetanib and cabozantinib, dose modifications were needed in response to adverse events.

Everolimus, an mTOR inhibitor, has been reported in a phase II trial to show antitumor activity in advanced MTC with the main toxicities being mucositis, fatigue and hypertriglyceridemia.^33^

Pre-clinical studies of interest include the suppression of RET transcription in a human MTC cell line (MTC TT) with the use of a natural product berberine.^34^ This led to cell cycle arrest and apoptosis. Another group investigated the use of an HSP90 inhibitor (AUY922) which has a role of regulating RET and so by inhibiting HSP90, this had led to MTC TT cell apoptosis and downregulation of the mitogen activated protein kinases (MAPK) and mTOR pathways.^35^

As research continues on the aberrant molecular pathways, it is expected that more inhibitors will be identified, and a number of novel small molecule inhibitors as single agent but also in combinations are currently under investigation.

## Management in Real Life

Patients with locally advanced or metastatic MTC until relatively recently were offered best supportive care but there are now treatment options with licensed TKIs. Careful consideration is required, howver, to achieve a balance between benefit and toxicity to a patient once started on one of these drugs. The aim should be to prolong life, prevent the onset or slow the worsening of symptoms whilst not significantly compromising quality of life. To date there is no robust evidence to show that the licensed TKIs prolong overall survival. Therefore, the decision to initiate therapy is not straight forward and is best made within the multi-disciplinary setting with full involvement of the patient and carer. Progression of disease clinically, radiologically and the development or imminent development of symptoms are factors taken into account. Once a decision to start therapy has been made, regular monitoring and adjustment of dose and supportive medications will enhance this balance and hence clinical benefit. In an effort to improve outcomes and combat the development of resistance, combination and sequential therapy regimens are currently under review in clinical trials.

In our Institution, patients are reviewed weekly in the first 4–6 weeks following initiation of treatment, and then follow up intervals are extended according to tolerance and response to the drug. Time is well spent educating the patient and carer prior to starting treatment, about the potential side effects and simple measures to avoid or lessen these. A clinical nurse specialist is the ideal contact for the patient to assist with this. At each review, careful attention is made to ensure that the side effects are manageable and treated early. We find the involvement and advice of specialist colleagues such as cardiologists and dermatologists invaluable in some cases where specific side effects are refractory to initial supportive measures. Not many patients remain on the full starting dose of the TKIs and dose modifications to ensure tolerability are essential to permit compliance over potentially long periods of time.

MTC is rare in children and experience of treatment of advanced MTC with systemic therapies is limited. Vandetanib has been used in the paediatric population and has shown similar responses and toxicities as those reported in adults.^36^

## Future Directions

Although we are equipped with two TKIs, which have both been shown to be effective in this rare cancer, the sequencing of the drugs is still unclear i.e., which drug should be used first. The patient cohort in the EXAM trial may represent a poorer prognostic group compared to the ZETA trial in view of the shorter PFS in the placebo arm and the requirement for documented progression of disease by RECIST criteria for entry into trial.^28,26^ However, it would be unwise to draw any conclusions regarding superiority of one drug over the other without a trial directly comparing both drugs.

The TKIs are not without toxicities and although both drugs have been approved at their maximum tolerated dose (140 mg cabozatinib and 300 mg vandetanib), the majority of patients need a dose reduction. Further studies are ongoing to assess the efficacy of starting at lower dose levels.

Data on overall survival is still awaited and further research needs to be made into the mechanisms of resistance and whether it would be beneficial and safe to use a combination of drugs.

Further studies need to be completed regarding the significance of RET mutation as a predictive biomarker. It has been suggested that M918T mutations in sporadic MTC lead to a higher response rate to TKIs compared to non-mutants. The question of whether the mutation status or molecular profile overall of individual tumours could be used as a biomarker to predict response or aid in drug selection, is still to be answered.

## Conclusion

MTC is a rare cancer and until data from the ZETA and EXAM trials,^26,27^ treatment for those with locally advanced or metastatic disease had been best supportive care. With the understanding of aberrant pathways in MTC and new targeted therapies, we may expect the continued development of targeted agents with the potential to improve outcomes for this population of patients.
